# Hepatic Arterial Infusion Pump Chemotherapy for Unresectable Intrahepatic Cholangiocarcinoma: A Systematic Review and Meta-Analysis

**DOI:** 10.1245/s10434-022-11439-x

**Published:** 2022-03-16

**Authors:** Jessica J. Holster, Marouan El Hassnaoui, Stijn Franssen, Jan N. M. IJzermans, Jeroen de Jonge, Bianca Mostert, Wojciech G. Polak, Roeland F. de Wilde, Marjolein Y. V. Homs, Bas Groot Koerkamp

**Affiliations:** 1 Department of Surgery, Erasmus MC Cancer Institute, Rotterdam, The Netherlands; 2 Department of Medical Oncology, Erasmus MC Cancer Institute, Rotterdam, The Netherlands

**Keywords:** Unresectable Intrahepatic Cholangiocarcinoma, Hepatic Arterial Infusion Pump (HAIP), Chemotherapy, Floxuridine, Biliary Tract Cancer

## Abstract

**Background:**

Patients with unresectable intrahepatic cholangiocarcinoma (iCCA) have poor survival. This systematic review describes the survival outcomes of hepatic arterial infusion pump (HAIP) chemotherapy with floxuridine for patients with unresectable iCCA.

**Patients and Methods:**

A literature search was conducted using the electronic databases PubMed, Medline (Ovid), Embase, Web of Science, Google Scholar, and Cochrane to find studies that reported data on the survival of patients with unresectable iCCA treated with HAIP chemotherapy using floxuridine. The quality of the studies was assessed using the Newcastle–Ottawa quality assessment Scale (NOS). Overall survival (OS) was the primary outcome measure, and progression-free survival (PFS), response rates, resection rates, and toxicity were defined as secondary outcome measures.

**Results:**

After removing duplicates, 661 publications were assessed, of which nine studies, representing a total of 478 patients, met the inclusion criteria. Three out of nine studies were phase II clinical trials, one study was a prospective dose-escalation study, and the remaining five studies were retrospective cohort studies. After accounting for overlapping cohorts, 154 unique patients were included for pooled analysis. The weighted median OS of patients with unresectable iCCA treated with HAIP chemotherapy with floxuridine was 29.0 months (range 25.0–39 months). The pooled 1-, 2-, 3-, and 5-year OS were 86.4, 55.5, 39.5, and 9.7%, respectively.

**Conclusion:**

HAIP chemotherapy with floxuridine for patients with unresectable iCCA was associated with a 3-year OS of 39.5%, which is favorable compared with systemic chemotherapy for which no 3-year survivors were reported in the Advanced Biliary Cancer (ABC) trials.

**Supplementary Information:**

The online version contains supplementary material available at 10.1245/s10434-022-11439-x.

Intrahepatic cholangiocarcinoma (iCCA) is a subtype of the biliary tract malignancies, distinguished by its intrahepatic origin proximal to the second-order biliary radicles. In recent years, there has been a worldwide increase in the incidence and mortality of iCCA.^1,2^ Patients with iCCA often remain asymptomatic for a long time, and are therefore hard to diagnose early.^3^ Consequently, the majority of patients have advanced disease at diagnosis and do not qualify for curative-intent surgery.^4,5^ The median overall survival (OS) of patients with unresectable iCCA who remain untreated is less than 5 months.^6^ Notably, about 70% of patients with unresectable iCCA die from progressive disease in the liver and subsequent liver failure, rather than from widespread metastatic disease.^7^ Systemic chemotherapy is the standard of care for patients with advanced biliary tract cancer, based on the Advanced Biliary Cancer (ABC)-02 randomized controlled trial. The median OS in this trial was 8.1 months for patients who received gemcitabine alone versus 11.7 months for patients who received both gemcitabine and cisplatin (HR 0.64, *p* < 0.001).^8^ A subgroup analysis was performed on the 34 patients who received gemcitabine-cisplatin for liver-only unresectable iCCA in the ABC trials.^9^ The median overall survival for patients with unresectable iCCA was 16.7 months, with no patients surviving beyond 3 years.

Because most patients die from progressive disease in the liver, hepatic arterial infusion pump (HAIP) chemotherapy with floxuridine is an attractive treatment option for unresectable iCCA.^10^ The rationale for HAIP chemotherapy is that iCCA relies mostly on arterial blood supply.^11,12^ Moreover, floxuridine, also known as FUDR, is characterized by its high first-pass effect; approximately 95% is directly metabolized in the liver. Hence, this allows for an up to 400-fold dose increase in subsequent intratumoral exposure compared with systemic treatment, with minimal systemic exposure and side effects.^13^

The aim of this systematic review was to investigate survival outcomes of HAIP chemotherapy with floxuridine in patients with unresectable iCCA.

## Patients and Methods

### Search Strategy and Selection Criteria

A literature search was conducted using the electronic databases PubMed, Medline (Ovid), Embase, Web of Science, Google Scholar, and Cochrane to find studies that describe the survival outcome of HAIP with floxuridine in patients with unresectable iCCA. The search strategies can be found in Supplementary Table S1. The last search was performed on 9 June 2021. We adhered to the Preferred Reporting Items for Systematic Reviews and Meta-Analyses (PRISMA) guidelines for reporting systematic reviews.^14^ The protocol of the study was registered on the International Prospective Register of Systematic Reviews, PROSPERO (CRD42020222821).

Observational studies and randomized controlled trials written in English that investigated the effect of HAIP with floxuridine, whether or not combined with concurrent systemic chemotherapy, in adults with unresectable iCCA were eligible for inclusion. Studies were excluded if they met one or more of the following criteria: (a) they did not treat patients with HAIP chemotherapy with floxuridine; (b) they included patients who received different therapies, without separately reporting outcomes of the patients treated with HAIP chemotherapy with floxuridine; (c) they did not report OS; (d) they included fewer than five patients with unresectable iCCA; and (e) the full text was not available.

### Data Extraction

Two authors (J.J.H., M.e.H.) independently assessed the title and abstract of all studies found with the literature search strategy and applied the inclusion and exclusion criteria to conclude whether the studies were eligible. If a study was potentially eligible, the two authors assessed the full text of the study. Disagreements were resolved with the help of a third author (S.F.). The following data were independently extracted from the included studies by the first and second authors: publication year, study design, research site, period of inclusion, treatment regimen, sample size, median follow-up, and primary and secondary outcomes. The primary outcome measure of our review was OS, expressed as a weighted median OS, and a pooled 1-, 2-, 3-, and 5-year OS. Secondary outcomes were progression-free survival (PFS), toxicity, response rates, and resection rates. Response rates are reported according to the Response Evaluation Criteria in Solid Tumors (RECIST).^15,16^

### Methodological Assessment

Two authors (J.J.H., M.e.H.) independently assessed the quality of the included studies. In case of disagreement, a third author (S.F.) was consulted for discussion. The Newcastle–Ottawa Scale (NOS) for assessing the quality of nonrandomized studies in meta-analyses was used to assess the quality of the included cohort and case-control studies (Supplementary Fig. S1).^17^ Studies that scored 3 or fewer points were considered as low quality, studies that scored 4–6 points as moderate quality, and studies that scored 7 points or higher as high-quality studies.

### Statistical Analysis

The weighted median OS was calculated for all unresectable iCCA patients in the included studies. The weighted estimate of median survival (*m*_*p*_) was derived with the help of the following formula:

1 $${\text{m}}_{{\text{p}}} { = }\left( {\mathop \sum \limits_{{\text{i = 1}}}^{{\text{k}}} \frac{{{\text{w}}_{{\text{i}}} }}{{{\text{m}}_{{\text{i}}} }}} \right)^{{{\text{{-}1}}}}$$

In this formula, *m*_*i*_ denotes the median survival in a study population *i* (with *i* ranging from 1 to *k*, where *k* is the number of included studies) and *w*_*i*_ refers to a study-specific weight function. The specific weight function in this study is the number of study participants divided by the total number of evaluable patients.^18,19^ The range of the weighted median OS is provided, since confidence intervals were not calculable. The 1-, 2-, 3-, and 5-year OS were pooled and presented as pooled proportions with 95% confidence interval. If the 1-, 2-, 3-, or 5-year OS were not mentioned by the authors of the studies, they were derived from the Kaplan–Meier curves where possible, otherwise the study was excluded from the subpart of the pooled analysis. The random-effects model described by DerSimonian and Laird (DL) was used, expecting heterogeneity of treatment effects between studies, assessed by the *I*^*2*^ statistic and the DL estimator for $\tau^{2}$ .^20^ Meta-analyses were conducted in the software program *R* version 4.1.0 (R Foundation for Statistical Computing, Vienna, Austria) using the software package “meta”. Sensitivity analyses were conducted to examine potential influences on the survival outcomes.

The secondary outcome PFS was also presented as a weighted median and calculated in the same manner as the weighted median OS. The secondary outcome response rates were presented as pooled proportions and calculated in the same manner as the pooled 1-, 2-, 3-, and 5-year OS. Publication bias was assessed with the Egger’s regression test and a funnel plot if > 10 studies were included.^21,22^ A *p*-value < 0.05 (two-tailed) was considered as statistically significant.

## Results

**Fig. 1:**
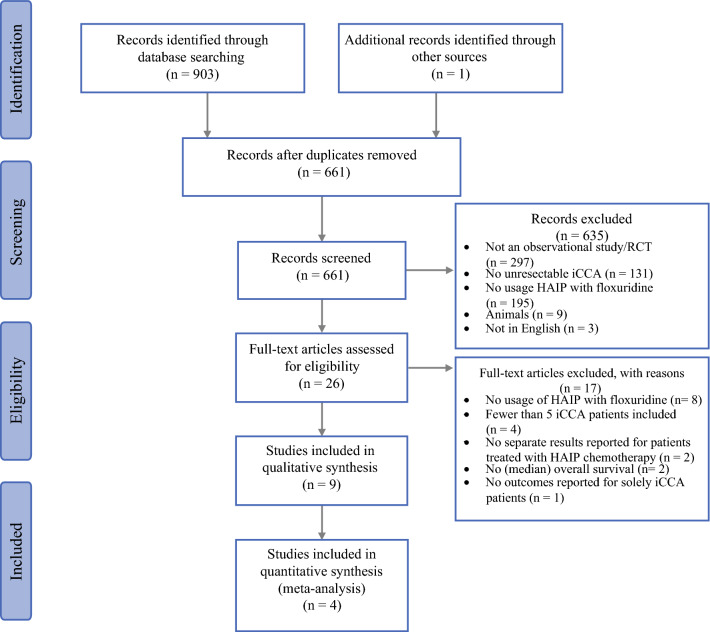
Preferred Reporting Items for Systematic Reviews and Meta-Analyses (PRISMA) flow diagram of the literature search

The literature search strategy resulted in 661 publications after removing duplicates (Fig. 1). After checking eligibility based on title and abstract, the full text of 26 articles was assessed. After assessment of the full text, nine studies were included.^23–31^ The reasons for exclusion after full-text assessment were: no usage of HAIP with floxuridine for iCCA (*n* = 8), fewer than five iCCA patients (*n* = 4), no separate results reported for patients treated with HAIP chemotherapy (*n* = 2), no (median) overall survival reported (*n* = 2), and no outcomes reported for solely iCCA patients (*n* = 1) (Supplementary Table S2).

### Characteristics of the Studies

The nine included studies are described in Table 1. Three out of the nine studies were phase II clinical trials, one study was a prospective dose-escalation study, and the remaining five studies were retrospective cohort studies. The sample sizes varied from 12 to 196 patients. The total sample size consisted of 478 patients. Several studies had overlapping cohorts, resulting in a sample size of 154 unique patients for pooled analysis. One study reported long-term follow-up of two phase II trials, which were also included in the qualitative synthesis.^24,30,31^ None of the patients had extrahepatic disease other than locoregional nodal disease at the start of treatment (i.e., lymph nodes in the portocaval and hilar regions), with one study including patients with limited and potentially resectable small lung nodules.^29^ The median follow-up time varied from 29.3 to 43.8 months. Across all studies, patients were included from December 1990 until June 2019. The research sites of the studies were mostly in the USA: Memorial Sloan Kettering Cancer Center (MSKCC, New York, USA), University of Pittsburgh Medical Center (UPMC, Pittsburgh, USA), and Washington University (St. Louis, USA). One study was performed at the University Hospital Zurich (USZ, Zürich, Switzerland) (Table 1).

**Table 1:** Characteristics of included studies

Author	Publication year	Study design	Site	Period of inclusion	Sample size, no.	Age, median (range), *years*	Sex, no. of men (%)
Jolissaint ^28^ *	2021	Retrospective cohort	MSKCC	2000–2018	196	62.0 (30.1–85.7)	116 (59.2)
Pietge ^29^	2021	Prospective, dose-escalation study	University Hospital Zurich	June 2012 to January 2016	12 ^a^	63.5 (33–72)	9 (75)
Cercek ^26^ *	2019	Phase II trial	MSKCC	May 2013 to June 2019	38	64 (39-81)	13 (34)
			Washington University in St. Louis	May 2013 to June 2019	10	63 (45–80)	NR
Wright ^27^	2017	Retrospective cohort	University of Pittsburgh Medical Center	January 2004 to June 2016	16	60.3 (12.3) ^f^	8 (50)
Konstantinidis ^25^ *	2016	Retrospective cohort	MSKCC	January 2000 to August 2012	78 ^b^	62 (30–84)	31 (40)
Konstantinidis ^24^ *	2014	Retrospective cohort	MSKCC	August 2003 to September 2009	44 ^c^	59 (13.2) ^f^	13 (30)
Kemeny ^31^ *	2011	Phase II trial	MSKCC	NR	22 ^d^	NR	10 (45.5)
Jarnagin ^30^ *	2009	Phase II trial	MSKCC	August 2003 to March 2007	34 ^e^	56.5 (30–85)	12 (35.3)
Endo ^23^ *	2008	Retrospective cohort	MSKCC	December 1990 to July 2006	28	NR	NR

Author	Prior treatment, no. (%)		Concurrent SYS, no. (%)	Tumor size, median (range), *cm*	Bilobar disease, no. (%)	Multifocal disease, no. (%)	Locoregional nodal disease, no. (%)	Median follow-up, months
	SYS	LR						
Jolissaint ^28^ *	NR	NR	142 (72.4)	8.6 (1.0–19.5)	NR	145 (74.0)	56 (28.6)	NR
Pietge ^29^	2 (17)	0 (0)	12 (100)	NR	NR	NR	4 (33)	43.8
Cercek ^26^ *	3 (8)	NR	38 (100)	8.3 (1.7–24.8)	25 (66)	21 (55)	18 (47)	30.5
	2 (20)	NR	10 (100)	NR	NR	7 (70)	2 (20)	NR
Wright ^27^	NR	NR	8 (50)	9.4 (4.1–19.2)	13 (81.3)	16 (100)	11 (68.8)	NR
Konstantinidis ^25^ *	NR	NR	78 (100)	9.4 (2.1–14.6)	61 (78)	51 (65)	0 (0)	NR
Konstantinidis ^24^ *	7 (16)	5 (11)	18 (41)	9.3 (2.1–16.4)	NR	14 (32)	NR	29.3
Kemeny ^31^ *	3 (13.6)	1 (4.5)	22 (100)	9 (1.1–16.4)	NR	15 (68.1)	NR	NR
Jarnagin ^30^ *	3 (8.8)	7 (20.6) ^g^	0 (0)	9.7 (2.7–18.1)	NR	21 (61.8)	NR	35
Endo ^23^ *	NR	NR	NR	NR	NR	NR	NR	NR

*SYS* Systemic therapy, *LR* locoregional treatment, *MSKCC* Memorial Sloan Kettering Cancer Center, *NR* not reported, *iCCA* intrahepatic cholangiocarcinoma, *HCC* hepatocellular carcinoma

^*^Studies with overlapping patient cohorts

^a^Nine patients with iCCA, two patients with hilar CCA, one patient with gallbladder cancer

^b^Including patients previously selected in Konstantinidis et al. (2014)

^c^Including the iCCA patients previously selected in Kemeny et al. and Jarnagin et al.

^d^Eighteen iCCA patients, 4 HCC patients

^e^Twenty-six iCCA patients, 8 HCC patients

^f^Age, mean (SD)

^g^Three patients were treated with more than one modality

Five studies reported data on prior treatment.^24,26,29–31^ The proportion of patients having received prior systemic chemotherapy ranged from 8 to 20% (Supplementary Table S3). Two out of five studies included patients who underwent prior locoregional treatment. In the study by Kemeny et al., one patient underwent prior ablation.^31^ In the study by Jarnagin et al., seven (21%) patients underwent prior locoregional treatment: five underwent ablation and two underwent resection.^30^

In seven studies, patients received concurrent systemic therapy and HAIP chemotherapy with floxuridine.^24–29,31^ In the study by Jolissaint et al., 142 (72%) patients received concurrent systemic chemotherapy: 58 received gemcitabine/oxaliplatin, 41 received irinotecan, and 27 received gemcitabine alone.^28^ In three studies, all patients received concurrent systemic chemotherapy; gemcitabine/cisplatin in the study by Pietge et al. and gemcitabine/oxaliplatin in the study by Cercek et al.^26,29^ In the study by Konstantinidis et al., a variety of systemic regimens were used.^25^ In the study by Wright et al., eight (50%) patients received concurrent systemic chemotherapy. The authors did not specify which chemotherapeutic agent or agents the patients received.^27^ In the study by Konstantinidis et al. and Kemeny et al., patients received concurrent systemic bevacizumab, respectively 18 (41%) and 22 (100%) patients.^24,31^

All included studies scored 4 or more points on the Newcastle–Ottawa Scale for assessing the quality of nonrandomized studies and were therefore ranked as studies of moderate or high quality (Supplementary Table S4). Hence, no subgroup analysis based on quality was performed. Testing for publication bias was not performed, because fewer than ten studies were included.

### Analysis of Overall Survival

The studies that included patients from MSKCC had overlapping periods of inclusion and therefore overlapping cohorts. The study with the longest period of inclusion and follow-up was Jolissaint et al.^28^ However, this study didn’t report OS for 59 (30.1%) patients receiving HAIP chemotherapy. The study by Konstantinidis et al., reporting the second longest follow-up of the patients from MSKCC, was used in pooled analyses instead.^25^ This study included all patients of three previous studies, including two phase II trials. After accounting for overlapping study populations, 154 patients remained for pooled analysis of OS.^25–27,29^

All nine studies reported median OS, except for the small separate cohort of Cercek et al. (Table 2).^26^ The weighted median OS calculated for the four most recent studies, with the exception of Jolissaint et al., representing 144 patients in total, was 29.0 months (range 25.0–39 months).^25–27,29^

**Table 2:** Survival rates

Author	Sample size, no.	Median OS, months	Confidence interval, 95%	1-year OS, %	2-year OS, %	3-year OS, %	5-year OS, %	Median PFS, months
Jolissaint ^28^	81 ^a^	24.9	20.3–29.6	85.2	46.9	21.0	4.9	NR
	56 ^b^	18.1	14.1–26.6	62.5	33.9	12.5	1.8	NR
Pietge ^29^	12	23.9	NR	75.0	41.7	33.3	0.0 ^*^	10.1
Cercek ^26^	38 ^c^	25.0	20.6–not reached	89.5	50.6 ^*^	43.7 ^*^	NR	11.8
	10 ^d^	NR	NR	70.0	40.0	NR	NR	12.8
Wright ^27^	16	39	32.7–51.3	86.9 ^*^	67.3 ^*^	50.5 ^*^	6.3	9.0
Konstantinidis ^25^	78	30.8	NR	86.7 ^*^	59.3	36.4 ^*^	22.0 ^*^	12.0
Konstantinidis ^24^	44	29.3	26.6–31.9	NR	NR	22.7	11.4	NR
Kemeny ^31^	18 ^e^	31.1	14.0–33.6	86.5 ^*^	62.0 ^*^	NR	NR	8.5
Jarnagin ^30^	26 ^f^	29.5	21.3–32.7 ^g^	88.2	67.6	29.4	NR	7.4
Endo ^23^	28	22	20–not reached	NR	NR	NR	NR	NR

*OS* Overall survival, *PFS* progression free survival, *NR* not reported

^*^Results derived from Kaplan–Meier curves

^a^N0 patients

^b^N1 patients

^c^MSKCC cohort

^d^Washington University in St. Louis cohort

^e^Eighteen iCCA patients, 4 HCC patients

^f^Twenty-six iCCA patients, 8 HCC patients

^g^Confidence interval derived from Kemeny et al.

**Fig. 2:**
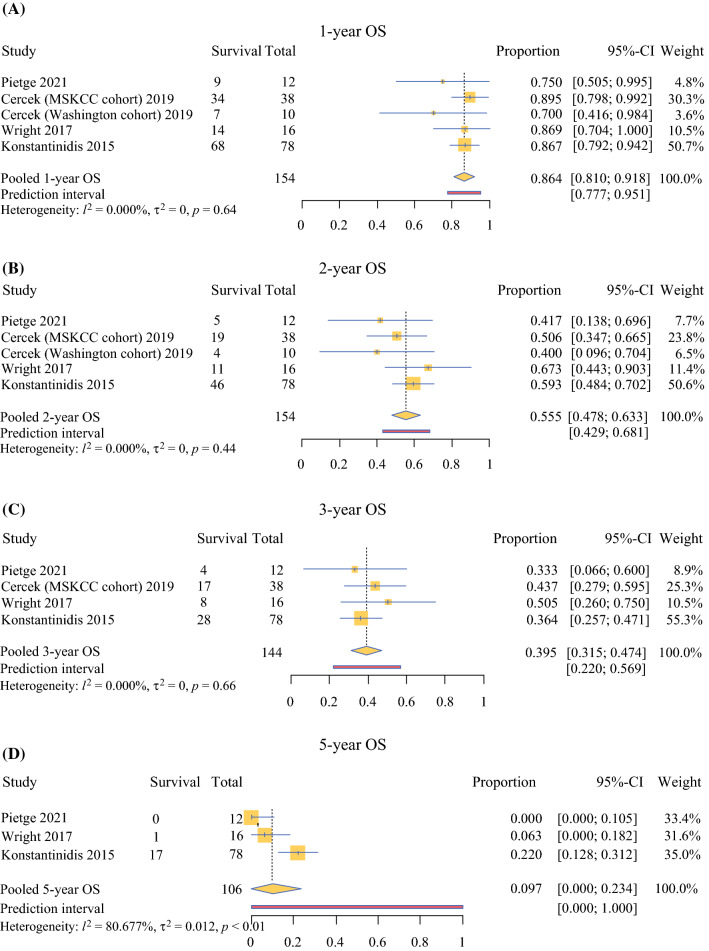
Forest plots showing **a** pooled 1-year OS, **b** pooled 2-year OS, **c** pooled 3-year OS, and **d** pooled 5-year OS with their 95%-CI in the random-effects model. *CI* Confidence Interval; *OS* Overall Survival

The pooled 1-year OS was 86.4% (95% CI 81.0–91.8%), 2-year OS 55.5% (95% CI 47.8–63.3%), and 3-year OS 39.5% (95% CI 31.5–47.4%). The pooled 5-year OS, based on three studies representing 106 patients, was 9.7% (95% CI 0.0–23.4%) (Fig. 2). For the small cohort (*n* = 10), Cercek et al. only reported data on 1- and 2-year OS, thus the weighted median OS and 3-year OS were calculated for 144 patients, and the 1- and 2-year OS for 154 patients (Table 2).

Sensitivity analysis including only the three phase II trials, representing 104 prospectively followed patients (92 with iCCA and 12 with HCC), found a weighted median OS of 27.8 months (range 25.0–31.1 months) and a pooled 1-, 2-, and 3-year OS of 87.5% (95% CI 81.2–93.8%), 57.7% (95% CI 47.0–68.3%), and 36.4% (95% CI 22.4–50.4%), respectively (Supplementary Fig. S2). None of the three phase II trials reported data on 5-year OS.

Median PFS was reported in six studies, ranging from 7.4 to 12.8 months (Table 2). The weighted median PFS, based on the four studies without overlapping patient cohorts, resulting in 154 individual patients, was 11.4 months (range 9.0–12.8 months).^25–27,29^

### Response and Resection Rates

Partial response (PR) was reported in six studies, ranging from 27% to 59%, and was above 50% in two phase II trials (Table 3). Stable disease (SD) was reported in five studies and ranged between 40 and 73%. Progressive disease (PD) was reported in five studies, ranging from 2.3 to 10.0%, where three patients in total were observed to have progression. The pooled PR was 52.7% (range 27.3–59.5%) based on the three most recent studies that reported PR, to avoid overlap between patient cohorts.^25,26,29^

**Table 3:** Response rates according to RECIST

Author	Sample size, no.	Version	Timing of measurement, months	PR, no. (%)	SD, no. (%)	PD, no. (%)
Jolissaint ^28^	196	NR	NR	NR	NR	NR
Pietge ^29^	11 ^a^	1.1	3	3 (27.3)	8 (72.7)	0 (0.0)
Cercek ^26^	38 ^b^	1.1	6	22 (57.9)	16 (42.1)	0 (0.0)
	10 ^c^	1.1	6	5 (50.0)	4 (40.0)	1 (10.0)
Wright ^27^	16	1.1 ^d^	6	NR	NR	NR
Konstantinidis ^25^	79 ^e^	1.1	NR	47 (59.5)	NR	NR
Konstantinidis ^24^	44	1.0	6	21 (47.7)	22 (50.0)	1 (2.3)
Kemeny ^31^	18 *	1.0	6	7 (38.8)	11 (61.1)	0 (0.0)
Jarnagin ^30^	26 *	1.0	6	14 (53.8)	11 (42.3)	1 (3.8)
Endo ^23^	28	NR	NR	NR	NR	NR

*RECIST* Response Evaluation Criteria in Solid Tumors*, PR* partial response, *SD* stable disease, *PD* progressive disease, *NR* not reported

^*^Without HCC patients

^a^One patient died after 6 weeks due to a pulmonary embolism

^b^MSKCC cohort

^c^Washington University in St. Louis cohort

^d^Used for determining clinical node positivity

^e^Seventy-eight, plus one unspecified patient

The rates of conversion to resection were reported in seven studies.^24–26,28–31^ In the study by Jolissaint et al., including the patients from the three phase II trials, 14 out of 196 patients underwent a curative intent resection after HAIP chemotherapy.^28^ In the study by Pietge et al., one patient underwent resection, achieving R1 resection status with eventual tumor progression 1 year postoperatively.^29^

### Toxicity and Adverse Events

Four studies reported on postoperative complications after placement of the intraarterial catheter and subcutaneous pump required for HAIP chemotherapy (Table 4). The number of postoperative complications varied between 7.9 and 83.3%. Most complications were reported in the study of Pietge et al., where 10 out of 12 patients experienced surgical complications: two had a Clavien–Dindo grade IIIB complication, one patient had a dislocation of the pump, and one patient developed a pump-related volvulus.^29^

**Table 4:** Toxicity and adverse events

Author	Toxicity			Postoperative complications	
	NCI CTCAE version	G3/G4 toxicity events, No.	Patients with G3/G4 toxicity, No. (%)	Complications, No. (%)	Type of complications
Jolissaint ^28^	NR	NR	NR	NR	NR
Pietge ^29^	4.03	32	NR	10 (83.3)	Seroma (*n* = 6), cholangitis (*n* = 1), anaphylaxis (*n* = 1), dislocation of the pump (*n* = 1), volvulus (*n* = 1)^a^
Cercek ^26^	4.0	79	NR	3 (7.9)	Gastroduodenal artery aneurysm (*n* = 2), infection at pump site (*n* = 1)
		16	NR	2 (20.0)	Gastroduodenal artery aneurysm (*n* = 1), extravasation HAI catheter (*n* = 1)
Wright ^27^	NR	NR	NR	NR	NR
Konstantinidis ^25^	3.0	NR	NR	NR	NR
Konstantinidis ^24^	3.0	NR	10 (22.7)	6 (13.6)	NR
Kemeny ^31^	3.0	26	NR	4 (18.2)	Wound infection (*n* = 1), fever (*n* = 1), acute pancreatitis (*n* = 1), infection at pump site (*n* = 1)
Jarnagin ^30^	3.0	NR	5 (14.7)	8 (23.5)	Wound infection (*n* = 3), pump misperfusion (*n* = 2), delirium (*n* = 1), SV tachycardia (*n* = 1)
Endo ^23^	NR	NR	NR	NR	NR

*NCI* National Cancer Institute, *CTCAE* Common Terminology Criteria for Adverse Events, *G3* grade 3 toxicity, *G4* grade 4 toxicity, *NR* not reported, *HAI* hepatic arterial infusion, *SV* Supraventricular

^a^Three patients had > one complication, the most severe is listed here

Five studies reported on toxicity due to chemotherapy (Table 4). All studies reporting toxicity used the National Cancer Institute (NCI) Common Toxicity Criteria version 3.0, except for Cercek et al. and Pietge et al., which used version 4.0.^26,29^ In the study by Pietge et al., 16 treatment-related grade 3/4 adverse events occurred in 12 patients. In the study by Cercek et al., seven (15%) patients had a grade 4 toxic or adverse event requiring discontinuation of HAIP chemotherapy, including one patient with an infection at the pump site and one patient with extravasation related to the HAI catheter. Four (11%) patients from the MSKCC cohort required biliary stents, two of which were due to chemotherapy-induced biliary sclerosis.^26^ In the study by Jarnagin et al., five (15%) patients had grade 3/4 toxicity and no patient developed biliary strictures.^30^ In the study by Kemeny et al., three (14%) patients required stents for biliary strictures, mostly related to the combination of floxuridine and bevacizumab. This trial was terminated early because of increased biliary toxicity.^24,30,31^

## Discussion

This review included nine studies representing 478 patients with unresectable iCCA who received HAIP chemotherapy with floxuridine, mostly with concomitant systemic chemotherapy. For the meta-analysis, 154 unique patients remained. The weighted median OS, calculated for 144 patients, was 29.0 months (range 25.0–39 months). The pooled 1-, 2-, 3-, and 5-year OS were 86.4, 55.5, 39.5, and 9.7%, respectively. The 3-year OS and 5-year OS were based on 144 and 106 patients, respectively. These results compare favorably to systemic chemotherapy alone, as the median OS of patients with unresectable iCCA who received gemcitabine with cisplatin in the ABC trials was 16.7 months.^9^ One-year OS in that study was 62.5%, 2-year OS was 24.5%, and no patient survived beyond 3 years.

Biliary obstruction and liver failure are the cause of death in most patients with unresectable iCCA.^7^ The objective of HAIP chemotherapy with floxuridine is to avoid or postpone disease progression in the liver. A small proportion of patients (less than 10%) may undergo a curative-intent resection after induction HAIP chemotherapy. Almost all patients with unresectable iCCA at presentation, however, will eventually develop distant metastases (i.e., in peritoneum, lung, and bone). Therefore, a resection after HAIP chemotherapy is unlikely to be curative and its role remains to be determined.

Besides HAIP, other locoregional treatments for unresectable iCCA have been studied.^32–34^ A systematic review of Yttrium-90 radioembolization reported a median OS of 15.5 months.^32^ A more recent large retrospective study of Yttrium-90 radioembolization found a median OS from first diagnosis of 29 months and a 3-year OS of 31%.^35^ However, the median OS after Yttrium was only 11 months, reflecting that most patients already had a long OS prior to Yttrium treatment. In a recent single-arm phase II trial, 41 patients received both first-line systemic gemcitabine with cisplatin and Yttrium-90 radioembolization. The median OS was 22 months, with a 1-year OS of 75% and a 2-year OS of 45%.^36^ This trial had a different patient population compared with the HAIP trial of Cercek et al., for example bilobar disease was less common (44% versus 66%), and determination of unresectability was different.^26^ The pooled OS results of HAIP chemotherapy with floxuridine compare favorably to Yttrium-90 radioembolization. However, no randomized comparison has been published. The SIRCCA trial is an ongoing randomized controlled trial (RCT) investigating the additional benefit of Yttrium-90 radioembolization to systemic chemotherapy; the accrual has been completed and results are expected in 2023–2024 (NCT02807181). A recent study found a median OS of 22.5 months after stereotactic body radiation therapy (SBRT) for unresectable iCCA in 37 patients, with 1- and 2-year OS of 69.7 and 46.5%, respectively.^37^ This treatment could be considered in patients with small lesions in whom a complete resection is not possible.

Several targeted treatments for iCCA are being investigated.^38^ About 13% of patients have isocitrate dehydrogenase 1 (*IDH1*) mutations and about 14% of patients have fibroblast growth factor receptor (*FGFR*) 2 fusions.^39,40^ In a phase III trial, patients with an *IDH1* mutation and advanced CCA who had progressed on previous therapy were randomized between ivosidenib and placebo. Cross over was allowed after progression. The progression-free survival (PFS) was better in the ivosidenib arm: 2.7 months compared with 1.4 months in the placebo arm (HR 0.37, one-sided *p* < 0.0001).^41^ OS did not statistically differ between the two groups (HR 0.79, one-sided *p* = 0.093).^42^ In a phase II trial, 61 patients with advanced iCCA with *FGFR2* alterations received infigratinib after first-line chemotherapy. The disease control rate was 75.4%, with a median PFS of 5.8 months.^43^ In an ongoing phase III trial, first-line pemigatinib is compared with systemic gemcitabine/cisplatin in patients with advanced cholangiocarcinoma with *FGFR2* rearrangements.^44^ The role of immunotherapy for iCCA has not yet been established, but is investigated in several clinical trials including the ongoing ABC-09 phase II trial and KEYNOTE-966 phase III trial (NCT03260712, NCT04003636). Approximately 2% of the patients with biliary tract cancer have high microsatellite instability (MSI) or mismatch repair (MMR) deficiency, for which small phase 2 studies showed that pembrolizumab is a treatment option.^45,46^ Targeted therapies and immunotherapy are emerging and seem to prove effective, with up to 50% of cholangiocarcinomas containing druggable mutations, amplifications, or fusions.^47^ In the current umbrella trial SAFIR ABC-10, molecular subtyping is used for precision treatment in patients with advanced cholangiocarcinoma.^48^ The optimal timing and sequence of these novel systemic treatments remains to be established.

One of the main limitations of this systematic review is the lack of randomized controlled trials. Three of the nine studies, however, were phase II clinical trials with a pooled 3-year OS of 36.4% compared with 0% after systemic chemotherapy in the ABC trials.^26,30,31^ Secondly, the number of studies and patients was small. The small, pooled sample size leads to less precision of the weighted and pooled OS estimates. Even the lower limits of the 95% confidence intervals, however, vastly exceed the 1-, 2-, and 3-year OS after systemic chemotherapy reported in the ABC trials. Though, it should be noted that the 5-year OS is an imprecise estimate given the few patients who remain alive up to that point and the presence of large between-study heterogeneity. Furthermore, patients were treated with different systemic chemotherapy regimens, which limits the homogeneity of the analyzed patients. Another limitation is the heterogeneity of reported postoperative complications and toxicity that is partly due to the small sample sizes of the studies. For these outcomes we refer to the paper describing complications and toxicity of more than 500 patients treated with HAIP chemotherapy by Allen et al.^49^ Lastly, external validation is needed, because most studies originated from the same institution (i.e., Memorial Sloan Kettering Cancer Center), making it difficult to translate HAIP chemotherapy as a more broadly applicable treatment modality. Currently, a phase II trial investigating OS after HAIP chemotherapy with floxuridine in patients with unresectable iCCA is ongoing in the Netherlands (NL8234).^50^ Also, an ongoing international randomized clinical trial (NCT04891289), initiated by MSKCC, is comparing systemic chemotherapy with or without HAIP with floxuridine for patients with unresectable iCCA. Future trials on HAIP chemotherapy should also investigate quality of life measures.

In conclusion, HAIP chemotherapy with floxuridine for patients with unresectable iCCA was associated with a favorable 3-year OS of 39.5% compared with systemic chemotherapy where no patients surviving beyond three years were observed in the ABC trials. Even though these results are quite impressive, external validation of these results is necessary besides a randomized controlled trial to optimally determine efficacy.

## Supplementary Information

Below is the link to the electronic supplementary material.

Supplementary file1 (DOCX 128 kb)
