# Supplementary material for: Hepatic Arterial Infusion Pump Chemotherapy for Unresectable Intrahepatic Cholangiocarcinoma: A Systematic Review and Meta-Analysis
Source: Ann Surg Oncol. 2022 Mar 16;29(9):5528–38. doi: 10.1245/s10434-022-11439-x (PMC9356931; doi:10.1245/s10434-022-11439-x)
Supplement: Supplementary file 1 — Supplementary file1 (DOCX 128 kb) [file 10434_2022_11439_MOESM1_ESM.docx]

*Supplementary Material*

**Hepatic Arterial Infusion Pump Chemotherapy for Unresectable Intrahepatic Cholangiocarcinoma: A Systematic Review and Meta-Analysis**

Jessica J. Holster, Marouan el Hassnaoui, Stijn Franssen, Jan N.M. IJzermans, Jeroen de Jonge, Bianca Mostert, Wojciech G. Polak, Roeland F. de Wilde, Marjolein Y.V. Homs and Bas Groot Koerkamp

|  |
| --- |

**FIG. S1** The Newcastle-Ottawa Scale (NOS) for assessing the quality of non-randomized studies in meta-analyses.

| **(a)**  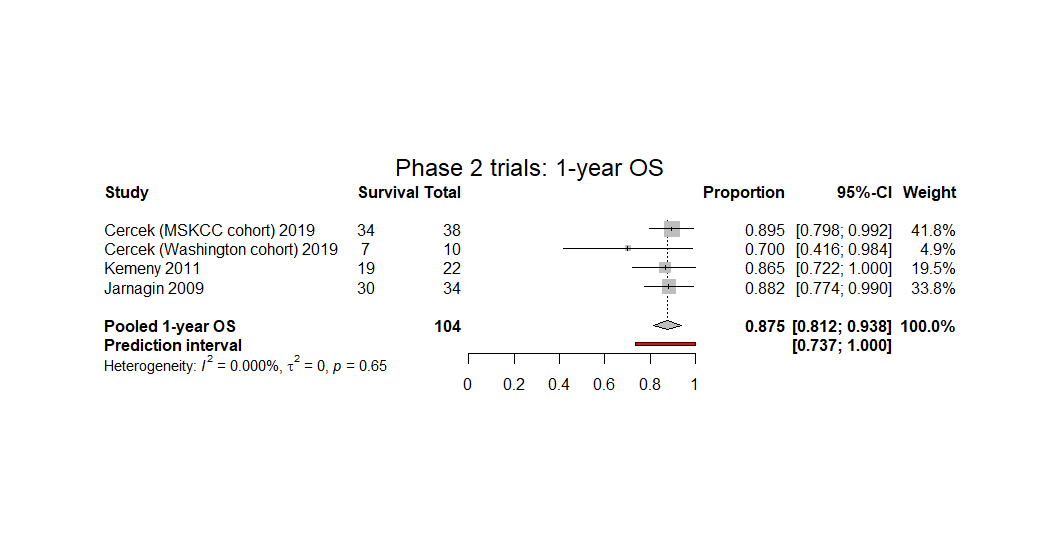  **(b)**  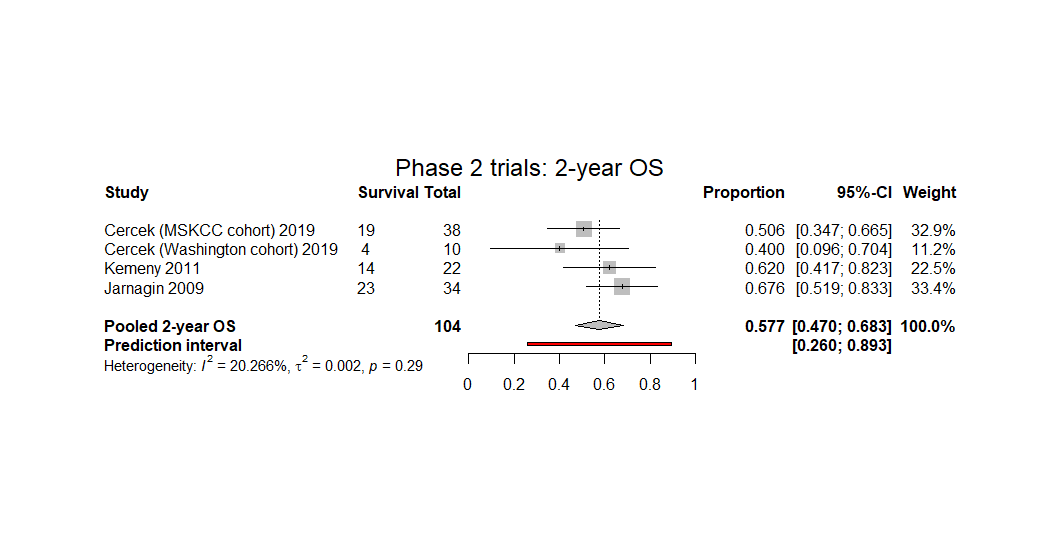  **(c)**  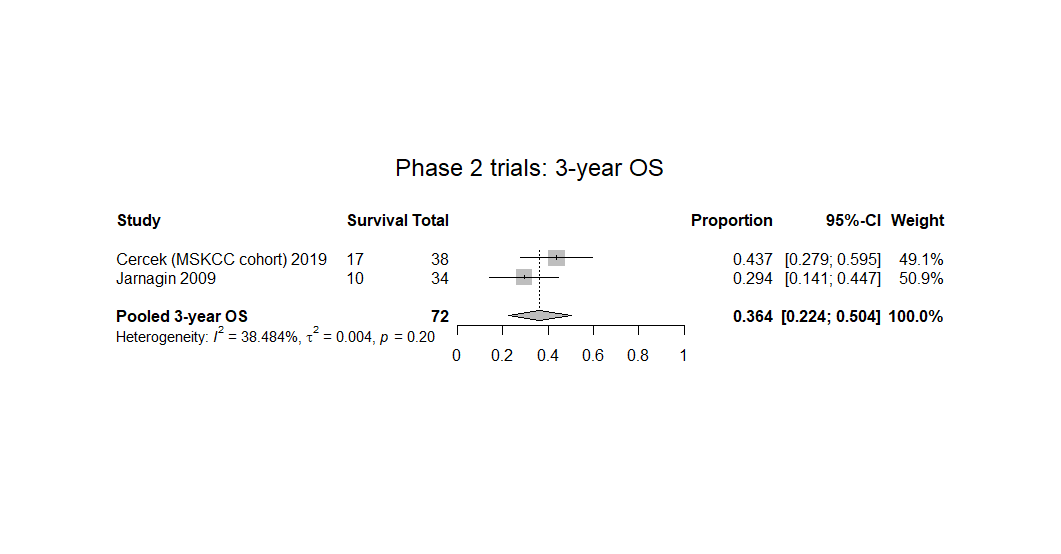 |
| --- |

**FIG. S2** Sensitivity analysis of the 1-, 2-, and 3-year OS identified in the 3 phase II trials. Forest plots showing **(a)** pooled 1-year OS, **(b)** pooled 2-year OS, and **(c)** pooled 3-year OS.

*OS Overall Survival; CI Confidence Interval.*

**TABLE S1** Search strategy for each database.

| **Database** | **Search terms** | **Results** |
| --- | --- | --- |
| PubMed | ("Cholangiocarcinoma"[Mesh] OR “Biliary tract neoplasms”[Mesh] OR “cholangiocarcinoma”[tiab] OR Biliary tract neoplasm* OR Biliary tract cancer* OR Bile duct cancer* OR Bile duct carcinoma* OR biliary tree carcinoma* OR carcinoma of the biliary tract* OR cholangiocellular carcinoma*) AND (("Floxuridine"[Mesh] OR floxuridine* OR FUDR* OR Fluorodeoxyuridine* OR 5-FUDR* OR 5-Fluorodeoxyuridine* OR chemotherapy*) AND (“Infusions, Intra-Arterial”[Mesh] OR infusion pump* OR HAIP* OR HAIT* OR hepatic arterial infusion* OR hepatic artery infusion* OR hepatic intra-arterial* OR Intra-arterial therapy* OR HAI chemotherapy*)) AND English[lang] | 220 |
| Embase | ('bile duct carcinoma'/de OR (cholangiocarcinoma* OR malignant-cholangioma* OR ((cholangiocellular* OR cholangiolar*) NEAR/3 (intrahepatic*) NEAR/6 (unresect*)) OR ((bile-tract* OR biliar* OR bile-duct*) NEAR/3 (carcinom* OR cancer* OR neoplasm* OR tumor* OR tumour*))):ab,ti,kw) AND ('floxuridine'/de OR 'implantable infusion pump'/de OR 'continuous infusion'/de OR (floxuridin* OR FUDR* OR Fluorodeoxyuridine* OR 5FUDR* OR 5Fluorodeoxyuridine* OR HAIP* OR HAIT* OR HAI-chemotherap* OR ((implant*) NEAR/3 (infus*) NEAR/3 (pump*)) OR ((continu*) NEAR/3 (infus*))):ab,ti,kw) | 330 |
| Medline(Ovid) | (exp "Biliary Tract Neoplasms"/ OR "Cholangiocarcinoma"/ OR (cholangiocarcinoma* OR malignant-cholangioma* OR ((cholangiocellular* OR cholangiolar*) ADJ3 (intrahepatic*) ADJ6 (unresect*)) OR ((bile-tract* OR biliar* OR bile-duct*) ADJ3 (carcinoma* OR cancer* OR neoplasm* OR tumor* OR tumour*))).ab,ti,kf.) AND ("Floxuridine"/ OR "Infusion Pumps, Implantable"/ OR ((floxuridine* OR FUDR* OR Fluorodeoxyuridine* OR 5FUDR* OR 5Fluorodeoxyuridine* OR HAIP* OR HAIT* OR HAI-chemotherap* OR ((implant*) ADJ3 (infus*) ADJ3 (pump*)) OR ((continu*) ADJ3 (infus*))).ab,ti,kf.)) | 143 |
| Web-of-Science | TS=(((cholangiocarcinoma* OR malignant-cholangioma* OR ((cholangiocellular* OR cholangiolar*) NEAR/2 (intrahepatic*) NEAR/5 (unresect*)) OR ((bile-tract* OR biliar* OR bile-duct*) NEAR/2 (carcinom* OR cancer* OR neoplasm* OR tumor* OR tumour*)))) AND ((floxuridin* OR FUDR* OR Fluorodeoxyuridine* OR 5FUDR* OR 5Fluorodeoxyuridine* OR HAIP* OR HAIT* OR HAI-chemotherap* OR ((implant*) NEAR/2 (infus*) NEAR/2 (pump*)) OR ((continu*) NEAR/2 (infus*))))) | 91 |
| Cochrane | ((cholangiocarcinoma* OR malignant-cholangioma* OR ((cholangiocellular* OR cholangiolar*) NEAR/3 (intrahepatic*) NEAR/6 (unresect*)) OR ((bile-tract* OR biliar* OR bile-duct*) NEAR/3 (carcinom* OR cancer* OR neoplasm* OR tumor* OR tumour*))):ab,ti) AND ((floxuridin* OR FUDR* OR Fluorodeoxyuridine* OR 5FUDR* OR 5Fluorodeoxyuridine* OR HAIP* OR HAIT* OR HAI-chemotherap* OR ((implant*) NEAR/3 (infus*) NEAR/3 (pump*)) OR ((continu*) NEAR/3 (infus*))):ab,ti) | 69 |
| Google Scholar  (50 top ranked) | unresectable cholangiocarcinoma floxuridine\|FUDR\|fluorodeoxyuridine\|5FUDR\|HAIP\|HAIT\|"HAI chemotherapy"\|"implantable infusion pump”\|"continuous infusion"  unresectable cholangiocarcinoma floxuridine\|FUDR\|fluorodeoxyuridine\|5FUDR\|HAIP\|HAIT\|'HAI chemotherapy'\|'implantable infusion pump'\|'continuous infusion' | 50 |

**TABLE S2** List of excluded articles based on full text with exclusion criteria.

| **References** | **Reasons** |
| --- | --- |
| ^1^ | <5 patients with intrahepatic cholangiocarcinoma |
| ^2^ | <5 patients with intrahepatic cholangiocarcinoma |
| ^3^ | <5 patients with intrahepatic cholangiocarcinoma |
| ^4^ | <5 patients with intrahepatic cholangiocarcinoma |
| ^5^ | Did not report (median) overall survival for cholangiocarcinoma |
| ^6^ | Did not report (median) overall survival for cholangiocarcinoma |
| ^7^ | No separate results reported for patients treated with HAIP chemotherapy |
| ^8^ | No separate results reported for patients treated with HAIP chemotherapy |
| ^9^ | No outcomes reported for solely iCCA patients |
| ^10^ | No usage of HAIP chemotherapy with floxuridine for (all) intrahepatic cholangiocarcinomas |
| ^11^ | No usage of HAIP chemotherapy with floxuridine for (all) intrahepatic cholangiocarcinomas |
| ^12^ | No usage of HAIP chemotherapy with floxuridine for (all) intrahepatic cholangiocarcinomas |
| ^13^ | No usage of HAIP chemotherapy with floxuridine for (all) intrahepatic cholangiocarcinomas |
| ^14^ | No usage of HAIP chemotherapy with floxuridine for (all) intrahepatic cholangiocarcinomas |
| ^15^ | No usage of HAIP chemotherapy with floxuridine for (all) intrahepatic cholangiocarcinomas |
| ^16^ | No usage of HAIP chemotherapy with floxuridine for (all) intrahepatic cholangiocarcinomas |
| ^17^ | No usage of HAIP chemotherapy with floxuridine for (all) intrahepatic cholangiocarcinomas |

**Abbreviation:** *HAIP* Hepatic arterial infusion pump; *iCCA* Intrahepatic cholangiocarcinoma.

**TABLE S3** Prior systemic chemotherapy.

| **Author** | **Sample size, No.** | **Prior systemic chemotherapy, No. (%)** | **Regimen** |
| --- | --- | --- | --- |
| Pietge ^18^ | 12 | 2 (16.7) | NR |
| Cercek ^19^ | 38 | 3 (7.9) | Gemcitabine/cisplatin (*n* = 2),  FOLFIRINOX (*n* = 1) |
|  | 10 | 2 (20.0) | Gemcitabine/cisplatin (*n* = 1), capecitabine/oxaliplatin (*n* = 1) |
| Konstantinidis ^20^ | 44 | 7 (15.9) | Gemcitabine/capecitabine (*n* = 2), gemcitabine/cisplatin (*n* = 1),  gemcitabine (*n* = 1), carboplatin/docetaxel/bevacizumab (*n* = 1),  >1 chemotherapy line (*n* = 2) |
| Kemeny ^21^ | 22 | 3 (13.6) | Gemcitabine/cisplatin (*n* = 1),  irinotecan/Xeloda (*n* = 1), Tarceva (*n* = 1) |
| Jarnagin ^22^ | 34 | 3 (8.8) | NR |

**Abbreviation:** *NR* Not reported.

**TABLE S4** Quality assessment of included studies with the Newcastle-Ottawa Scale (NOS).

|  | **Selection** |  |  |  | **Comparability** | **Outcome** |  |  |  |
| --- | --- | --- | --- | --- | --- | --- | --- | --- | --- |
| **Author** | **1** | **2** | **3** | **4** | **1** | **1** | **2** | **3** | **Total** |
| Jolissaint ^23^ | b | a | a | a | - | b | a | d | 6 |
| Pietge ^18^ | b | NA | a | a | - | b | a | a | 6 |
| Cercek ^19^ | b | NA | a | a | NA | b | a | a | 6 |
| Wright ^24^ | b | NA | a | a | b | b | a | d | 6 |
| Konstantinidis ^25^ | b | NA | a | a | NA | b | a | a | 6 |
| Konstantinidis ^20^ | b | NA | a | a | NA | b | a | a | 6 |
| Kemeny ^21^ | b | a | a | a | a,b | b | a | d | 8 |
| Jarnagin ^22^ | b | NA | a | a | NA | b | a | a | 6 |
| Endo ^26^ | b | c | a | a | - | b | a | d | 5 |

**Abbreviation:** *NA* Not applicable.

**REFERENCES**

1. Lawrence TS, Dworzanin LM, Walker-Andrews SC, et al. Treatment of cancers involving the liver and porta hepatis with external beam irradiation and intraarterial hepatic fluorodeoxyuridine. *Int J Radiat Oncol Biol Phys*. 1991;20(3):555-561.

2. Seeger J, Woodcock TM, Blumenreich MS, Richardson JD. Hepatic perfusion with FUdR utilizing an implantable system in patients with liver primary cancer or metastatic cancer confined to the liver. *Cancer Invest*. 1989;7(1):1-6.

3. Atiq OT, Kemeny N, Niedzwiecki D, Botet J. Treatment of unresectable primary liver cancer with intrahepatic fluorodeoxyuridine and mitomycin C through an implantable pump. *Cancer*. 1992;69(4):920-924.

4. Magge D, Choudry HA, Zeh HJ,3rd, et al. Outcome analysis of a decade-long experience of isolated hepatic perfusion for unresectable liver metastases at a single institution. *Ann Surg*. 2014;259(5):953-959.

5. Robertson JM, Lawrence TS, Dworzanin LM, et al. Treatment of primary hepatobiliary cancers with conformal radiation therapy and regional chemotherapy. *J Clin Oncol*. 1993;11(7):1286-1293.

6. Brajcich BC, Bentrem DJ, Yang AD, et al. Short-term risk of performing concurrent procedures with hepatic artery infusion pump placement. *Ann Surg Oncol*. 2020;27(13):5098-5106.

7. Ben-Josef E, Normolle D, Ensminger WD, et al. Phase II trial of high-dose conformal radiation therapy with concurrent hepatic artery floxuridine for unresectable intrahepatic malignancies. *J Clin Oncol*. 2005;23(34):8739-8747.

8. Robertson JM, McGinn CJ, Walker S, et al. A phase I trial of hepatic arterial bromodeoxyuridine and conformal radiation therapy for patients with primary hepatobiliary cancers or colorectal liver metastases. *Int J Radiat Oncol Biol Phys*. 1997;39(5):1087-1092.

9. Fowler K, Saad NE, Brunt E, et al. Biphenotypic primary liver carcinomas: Assessing outcomes of hepatic directed therapy. *Ann Surg Oncol*. 2015;22(13):4130-4137.

10. Spolverato G, Kim Y, Alexandrescu S, et al. Management and outcomes of patients with recurrent intrahepatic cholangiocarcinoma following previous curative-intent surgical resection. *Ann Surg Oncol*. 2016;23(1):235-243.

11. Melichar B, Voboril Z, Dvorák J, Ferko A, Rozkos T, Krajina A. Hepatic arterial infusion for biliary tract carcinoma: Single-center experience. *Anticancer Res*. 2013;33(3):1201-1207.

12. Kim JH, Yoon HK, Sung KB, et al. Transcatheter arterial chemoembolization or chemoinfusion for unresectable intrahepatic cholangiocarcinoma: Clinical efficacy and factors influencing outcomes. *Cancer*. 2008;113(7):1614-1622.

13. Hamada A, Yamakado K, Nakatsuka A, Takaki H, Takeda K. Clinical utility of coaxial reservoir system for hepatic arterial infusion chemotherapy. *J Vasc Interv Radiol*. 2007;18(10):1258-1263.

14. Cady B. Hepatic arterial patency and complications after catheterization for infusion chemotherapy. *Ann Surg*. 1973;178(2):156-161.

15. Reed ML, Vaitkevicius VK, Al-Sarraf M, et al. The practicality of chronic hepatic artery infusion therapy of primary and metastatic hepatic malignancies: Ten-year results of 124 patients in a prospective protocol. *Cancer*. 1981;47(2):402-409.

16. Gou Q, Wu L, Cui W, et al. Stent placement combined with intraluminal radiofrequency ablation and hepatic arterial infusion chemotherapy for advanced biliary tract cancers with biliary obstruction: A multicentre, retrospective, controlled study. *Eur Radiol*. 2021.

17. Shirono T, Niizeki T, Iwamoto H, et al. Therapeutic outcomes and prognostic factors of unresectable intrahepatic cholangiocarcinoma: A data mining analysis. *J Clin Med*. 2021;10(5):987. doi: 10.3390/jcm10050987.

18. Pietge H, Sánchez-Velázquez P, Akhoundova D, et al. Combination of HAI-FUDR and systemic gemcitabine and cisplatin in unresectable cholangiocarcinoma: A dose finding single center study. *Oncology*. 2021;99(5):300-309.

19. Cercek A, Boerner T, Tan BR, et al. Assessment of hepatic arterial infusion of floxuridine in combination with systemic gemcitabine and oxaliplatin in patients with unresectable intrahepatic cholangiocarcinoma: A phase 2 clinical trial. *JAMA Oncol*. 2020;6(1):60-67.

20. Konstantinidis IT, Do RK, Gultekin DH, et al. Regional chemotherapy for unresectable intrahepatic cholangiocarcinoma: A potential role for dynamic magnetic resonance imaging as an imaging biomarker and a survival update from two prospective clinical trials. *Ann Surg Oncol*. 2014;21(8):2675-2683.

21. Kemeny NE, Schwartz L, Gönen M, et al. Treating primary liver cancer with hepatic arterial infusion of floxuridine and dexamethasone: Does the addition of systemic bevacizumab improve results? *Oncology*. 2011;80(3-4):153-159.

22. Jarnagin WR, Schwartz LH, Gultekin DH, et al. Regional chemotherapy for unresectable primary liver cancer: Results of a phase II clinical trial and assessment of DCE-MRI as a biomarker of survival. *Ann Oncol*. 2009;20(9):1589-1595.

23. Jolissaint JS, Soares KC, Seier KP, et al. Intrahepatic cholangiocarcinoma with lymph node metastasis: Treatment-related outcomes and the role of tumor genomics in patient selection. *Clin Cancer Res*. 2021.

24. Wright GP, Perkins S, Jones H, et al. Surgical resection does not improve survival in multifocal intrahepatic cholangiocarcinoma: A comparison of surgical resection with intra-arterial therapies. *Ann Surg Oncol*. 2018;25(1):83-90.

25. Konstantinidis IT, Groot Koerkamp B, Do RK, et al. Unresectable intrahepatic cholangiocarcinoma: Systemic plus hepatic arterial infusion chemotherapy is associated with longer survival in comparison with systemic chemotherapy alone. *Cancer*. 2016;122(5):758-765.

26. Endo I, Gonen M, Yopp AC, et al. Intrahepatic cholangiocarcinoma: Rising frequency, improved survival, and determinants of outcome after resection. *Ann Surg*. 2008;248(1):84-96.
